# Notes and News

**Published:** 1887-10-29

**Authors:** 


### Notes and News.

Collected and Communicated.

The wards opened by the Princess of Wales in the National Hospital for the Paralysed and Epileptic, Bloomsbury, eighteen months ago, are stated to be now ready for occupation. This raises the number of available beds to 180.

The collection at Hawarden Church on behalf of the Chester General Infirmary amounted to £10 1s.

The Archbishop of Canterbury preached last Sunday at the parish church, Bromley, on the occasion of the annual church parade of Friendly Societies, on behalf of the funds of the Bromley Cottage Hospital.

Two deaths from small-pox are reported from Perth—one in the Fever Hospital and one at the General Infirmary.

At the annual meeting of the managers of the Bridgewater Infirmary held last week, the secretary was able to report a balance in the hands of the treasurer from last account of nearly £300.

The opinion prevalent in England, that milk is frequently a vehicle for the conveyance of epidemic diseases, and especially of scarlet fever, does not appear to be shared by medical authorities in India. According to the official sanitary report just published, the milk drinkers generally appear to have escaped fever, whilst those who do not drink milk have been largely affected by it.

The whale captured at Tilbury Fort on the 19th inst. will doubtless attract numerous visitors, very few of whom will realise that the great cetacean is a not very remote relative of the human race, similar in vital characteristics to the other mammalia, though popular phraseology, judging it, in proverbial fashion, by "the company it keeps," confounds it with the fishes among whom it dwells and on whom it preys. The unexpected visitor is no despicable one in point of size. It measures 35 ft. 4 in. in length, its mouth is 6 ft. wide ; its girth round the shoulders is 18 ft. 6 in., and its tail is 8 ft. across. Its total weight is 6 tons 5 cwts. As the proceeds of the exhibition of it are to be given to the Gravesend Hospital, we hope the whale will be, in playbill phrase, a great attraction.

The next evening meeting of The Hospitals Association will be held on Wednesday, 9th November, at 8 p.m. A paper will be read by Mr. Keith D. Young, F.R.I.B.A., on "Hospital Construction," and a discussion will follow. Sir Douglas Galton, K.C.B., will preside at the meeting, which will be held in the Board-room of the Middlesex Hospital, Berners Street, Oxford Street, kindly lent for the occasion by the weekly Board. Cards of admission can be obtained on application to the Secretary, The Hospitals Association, Norfolk House, Norfolk Street, W.C.

A Hospital Secretary writes :—"A Mr. F. J. Henderson, giving the address Charities' Central Collecting Agency, Bank of England Branch, Newcastle-on-Tyne, is applying to the London Charities for information as to their subscriptions. I understand that he proposes that subscriptions shall be paid through him at a cost of two-and-a-half per cent. Subscribers who wish to send their subscriptions through an agent can find no better one than their own bankers, who will pay the money over to the Charities without any cost whatever. I have referred Mr. Henderson to The Hospitals Association."

Hospital officials who may be waited upon by a benevolent stranger professing to represent the editor of a daily paper, and wishing to find out the depth of their necessities in order that the charitable editor may do what he can to relieve them, are warned against too implicitly trusting stamps, paper, and other trifles to the care of their amiable visitor.

In the early part of the week before last a house-to-house collection was made at Sutton Bridge on behalf of the Wisbeach and Lynn Hospitals, the total amount realised being about £5. The population of the Sutton sanitary district is considerably over 2,000. It may be hoped that the people, when they hear of the amount gathered from them on behalf of such a benevolent object, will have the grace to be ashamed of themselves.

Whitstable-on-Sea, with a population scarcely double that of Sutton Bridge, has also been making an effort on behalf of its hospital. As the result, the very handsome sum of £55 12s. 3d., after the deduction of all expenses, has been handed over to the hospital treasurer. This is an example well worth following.

The local treasurer at Berwick-on-Tweed has remitted to the Edinburgh Royal Infirmary the sum of £50 17s. 8d. The Edinburgh Infirmary gives accommodation to persons from all parts of Scotland and the North of England, and there are in many small towns and villages local associations and treasurers, which annually collect considerable sums on behalf of the institution. The people in those towns and villages feel that since they have the great advantage of sending serious cases to the hospital, they should also have the privilege of contributing to its funds, and they do so very liberally. Vast numbers of the patients who throng the London hospitals are likewise drawn from towns and villages more or less remote, and it is eminently desirable, both in the interests of the hospitals and those towns and villages which share in their benefits, that similar local societies with treasurers should be formed, with the view of bringing those towns into sympathetic and practical relationship with the hospitals. The Hospitals Association, working on the lines of the Edinburgh Infirmary, is making an effort to promote the establishment of such local hospital societies in all parts of England, and it is eminently desirable that the clergy and other ministers of religion and country practitioners should render assistance in the carrying out of an object so desirable and in every way advantageous to all parties.

THE following vacancies are announced this week, the amount of the salary being placed in each case after the name of the institution :—

**Honorary Medical Officers.**

North London Consumption Hospital. Physician.—University Graduate.
St. Thomas's Hospital.—Obstetric Physician.
Sussex County Hospital, Brighton. Assistant Physician.—University Graduate.
Taunton and Somerset Hospital.—Surgeon, F. or M.R.C.S.

**Resident Medical Officers.**

Birmingham Dispensary. Surgeon, doubly-qualified.—£150.
Blackburn Infirmary. Junior House-Surgeon, doubly-qualified.—£30.
Brighton County Hospital. Assistant House-Surgeon, doubly-qualified.—No salary.
Burnley Victoria Hospital. £80.—Hon. Sec., 16, Nicholas Street, Burnley.
Central London Ophthalmic Hospital. House-Surgeon.
Chelsea Dispensary. House-Surgeon and Secretary, doubly-qualified.—£95.
Liverpool Infirmary for Children. Assistant House-Surgeon.—No salary.
Lincoln Friendly Societies Dispensary. Doubly-qualified.—£200.
Maidstone Hospital. House-Surgeon, registered.—£150.
Rotherham Hospital. Assistant to House-Surgeon.—No salary.
Royal Hospital for Diseases of the Chest. Junior House Physician.—£50.
Scarborough Hospital. Assistant House-Surgeon, qualified.—£50.
Taunton and Somerset Hospital. House-Surgeon.—£100.
Winchester County Hospital. House-Surgeon, doubly-qualified.—£100.

**Non-resident Medical Officers.**

Evelina Hospital. Registrar and Chloroformist.—£30.
Vestry of St. Mary, Newington. Medical Officer of Health.—£300.

**Chaplain.**

Taunton and Somerset Hospital. In Priest's Orders.

**Matron.**

Manchester Hospital for Incurables.—£60.

**Trained Nurses.**

Bradford Eye and Ear Hospital.—£15.
Brompton Consumption Hospital. Two.
Bristol Royal Infirmary. Assistant Nurses.—£16 to £18.
City of London Hospital for Diseases of the Chest.—£20.
Hull Nurses' Home. Several.
Nursing Institute, Northampton. Several.
Surbiton Cottage Hospital. Assistant.—£25.
Victoria Hospital, Burnley. Head Nurse, Male Ward.—£26.
Workhouse Nursing Association. Several.

**Probationers.**

Ancoats Hospital, Manchester.—One.
Bristol Royal Infirmary. Several.
Royal Hospital for Diseases of the Chest. Lady Probationer.

---

THE total increase of £630 8s. 1d. in the income of the Sunderland and Bishopwearmouth Infirmary as compared with last year, is one of those gratifying facts which it is a pleasure to make known. The scale so frequently dips the other way that one is disposed to ask how it is that Sunderland has proved an exception to the rule. There is also a slight increase of expenditure, which, however, does not approach the amount by which the income is increased. We congratulate the secretary and managers on a very prosperous year.

---

ONE may have too much of a good thing. Irrigation is a very good thing for a hot and dry country, but unless it is accompanied by adequate drainage it brings unexpected evils in its train. This has been shown at a meeting for sanitary inquiry held in India, where it was explained that a great part of the fever that yearly carries off a considerable portion of the population was due to the moisture of the sub-soil, brought about by the network of canals that covers the land. Formerly, when the upland slopes were dry and barren, imperfectly moistened by bags of water hoisted up from well or river to a height of from thirty to sixty feet, people built their towns in the green shady valleys. Now that an abundant water-supply has transformed the barren uplands into fertile fields, the valleys have become morasses, and the towns hot-beds of fever, which are gradually becoming depopulated and deserted. Surely it is possible for modern intelligence and ingenuity to devise a scheme by which the gain of one district shall not entail ruin and death on another.

THE directors of the Perth Infirmary have replied to the charges brought by Dr. Simpson, the Medical Officer of Health for the city, against the infirmary management at the present crisis. The principal point in the reply is to the effect that, as Dr. Simpson is a comparatively young man and Dr. Stirling will soon be an old one, the former should have behaved with more deference to his professional senior. That is very well so far as it goes, but cannot be said to be a business-like way of dealing with the question.

---

AT a recent inquest on the body of an infant the cause of death was stated to be debility from want of proper feeding. The child had been fed on condensed milk, which the coroner said was absolutely poisonous to many children. This may seem to be very strong language, and condensed milk is too useful in many places—on board ship, and in towns where the milk is diluted frequently with impure water—to be readily banished from among our articles of diet. But at best these prepared foods—condensed milk, tinned meat, preserved vegetables—are only substitutes for fresh food-stuffs, and however useful in emergencies, should only be used when the latter cannot be obtained. At the same time it may be noted that some of the healthiest children have been brought up entirely on condensed milk.

---

ENGLAND has too many doctors—so many, that she can use them up and kill them with as much certainty of getting more as if they were flies in summer. Russia, on the other hand, has too few—little more than 5,000 to a population of 100,000,000. What is the result of this lack of medical men? A very grievous one,—that in Russia about 2,800,000 people die every year, nearly half of whom, in the opinion of the Vice-Director of the Medicinal Department at St. Petersburg, could be saved if they had proper medical attendance and improved sanitary conditions. In some districts the death-rate is 80 for every 1,000 inhabitants, and of this mortality from 59 to 79 per cent. is that of children. Such facts as these ought to be a convincing reply to those opinionated people who "don't believe in doctors," and think they would live quite as long and healthily without the physician's aid. Let them go to Russia and try.

---

IN view of the present epidemic of scarlet fever, it seems opportune to speak of the desirability of the presence of infectious diseases being notified to the authorities. The relatives or attendants of sufferers should be compelled to give immediate information of the presence of any infectious case. Objections have been raised, indeed, to the demand for the necessary Act of Parliament to ensure this notification, on the ground that it would destroy the confidential relations existing between doctor and patient, and that many people would be tempted to evade the conditions of the Act by not calling in a medical man. The first of these objections may be speedily dismissed. The confidence existing between doctor and patient seldom has reference to the nature of his illness, and to notify the existence of it to proper authorities is not, as some people seem to think, identical with blazoning its cause and symptoms to the world at large. The second complaint is better based; but Dr. Tomkins, the Medical Officer of Health for the borough of Leicester, in which town compulsory notification has been in force for eight years, states, in a letter to the *Times*, that his experience proves such a fear to be unnecessary. After all, the health of the public at large must be considered before the feelings of individuals, and there seems little doubt that this would be best consulted by the notification of infectious diseases being made compulsory on the parents and medical attendant, and any neglect or evasion being punished with some severity.

The Guernsey Convalescent Home at Luck's All is now opened for the reception of poor persons needing change of air. The charge is 7s. 6d. a week. The air of Guernsey is very suitable for a large class of convalescents, and we are glad to see that the home is to be made extensively available.

---

The Venus of Milo is the acknowledged Queen of Beauty of the world. Standing majestic in her stately grace, she receives homage from visitors from every quarter of the globe, many of them natives of countries undiscovered when the Greek sculptor carved his masterpiece in marble; and discontented artists exclaim that there are not to be found nowadays women of such pure regularity of feature. Professor Karl Hasse has, however, measured exactly the dimensions and proportions of the Venus's head, and finds that her features are not regular after all. The left ear is higher than the right, and the left side of the face is altogether higher and nearer to the middle line than the other; even the skull is broader on the left side than on the right. It may be some consolation to ladies who deplore the lack of classic perfectness in their faces, to learn that the features of even this gem of classic art are not "icily regular," with the possibility of being also, like the Laureate's heroine, "splendidly null." There must be some charm in irregularity when art as well as nature achieves beautiful results by its aid.

---

Our contemporary, the *British Medical Journal*, did good service last week by commending The Hospitals Association to the favourable attention of its readers. It is a kindly notice, and the explanation of Sir Andrew Clark's scheme to increase the funds of all the hospitals is well timed and helpful. Under the editorship of Mr. Ernest Hart the *British Medical Journal* has helped forward many movements which have greatly benefited large masses of the people. The Home Hospitals Association for Paying Patients, The National Health Society, Lord Brabazon's Association for providing Open Spaces, The Medical Annuity and Sick Fund, The Medical Benevolent Fund, and the Epsom Benevolent College, are all indebted to the *British Medical Journal* for generous and consistent support. We do not think it is too much to say that the columns of the *British Medical Journal* have never been closed by Mr. Ernest Hart when the aims and claims of any really deserving movement have had to be urged. It is an editor's function at times to blame as well as to praise, and it would be difficult to say which policy has done most good to the institutions referred to, so judicious has been the course taken by the conductors of the journal of the British Medical Association.

---

A correspondent, who signs himself "Manual Labour," addresses some very pointed remarks to the editor of the *Blackpool Gazette* on the subject of the Victoria Hospital. He thus writes:—"So the public meetings, the speeches, the vows last spring, the Lord Mayor's visit, the processions, the banquets, the balls, the ceremonies, the honours, and the myriad of other great events have resulted in the hospital coming down to a penny subscription list. Oh, fie! Dr. Cocker. Fie! ye feeders on the North Pier; ye swiggers of champagne and spouters of long mutual butterings. Oh, worldly ones, how soon you have forgotten the virtuous resolves which made your spotless fronts to swell while the champagne was trickling down your windpipes! A penny subscription list, Aldermen and gentlemen. A penny subscription list, Mesdames Pink Parasol and White Lace Skirt. A penny subscription list, ye swashbucklers of the North Pier. Where are our generous men of money, our magnanimous promisers and our loud spouters, now? Come out, now that the penny subscription list is going round, and build the hospital, and show you meant all you said when there were feeds and fun to entice you."

---

Street collections on behalf of hospitals do not appear to be growing in popularity. At Barnstable, which is a town of considerable importance, the amount collected did not exceed £2. This is stated to be slightly less than last year's contribution. Clearly the Barnstable people do not wear their charitable hearts upon their sleeves for hospital daws to peck at.

---

Lord Northbourne has given a site of two acres for the erection of a Jubilee hospital at Gateshead. He has also made known his intention of making a donation of £200 towards the building fund,—a sum which, when added to previous amounts, makes a total of £900 contributed by the Northbourne family. The total cost of the building will be little over £2,000.

---

The names are still being registered at Norfolk House of nurses and officials who are anxious to join the Pension Fund. Sir Andrew Clark has, we believe, communicated with the Queen, and all who desire to avail themselves of the advantages of the National Pension Fund should send their names to Mr. Collins, at Norfolk House, without a moment's delay. Any objections, suggested difficulties, or inquiries should be addressed to Mr. Burdett, at The Lodge, Porchester Square, W.

---

Mr. Thomas Blair, the General Manager of the Leeds Infirmary, is anxious to obtain for his Board all the information possible about the most suitable bedstead, whether iron or wood, which experience proves to be the best adapted for use in a small hospital in the country, where semi-convalescent and chronic patients will be treated. He will be glad if any hospital secretary will give him the benefit of the experience of his institution, and acquaint him whether (1) wood or iron bedsteads are in use at each institution supplying the information; (2) whether they are found satisfactory; and (3) which form of spring-mattress is the best. The information at the disposal of The Hospitals Association shows that the Bristol bedstead has a high reputation, whilst the wire-wove mattresses of Messrs. Chorlton and Dugdale are excellent, and the Lawson-Tait bedstead is being tried experimentally in some hospitals. Howe's spring-beds offer many advantages to people in average health and to convalescents.

---

Mr. Thomas Ryan, the secretary of St. Mary's Hospital, calls our attention to an error in the report of the proceedings at the Pension Fund meeting on Wednesday, the 12th inst., where it should have been stated that the motion of Lord Sandhurst was seconded by Mr. Ryan. Mr. Ryan further wishes it to be understood that the chief reason he gave for thinking that the public and the hospitals should be called upon to contribute to an endowment fund, was that very many nurses are widows with children to support, and many more have aged parents or other relatives whom they feel bound to assist, whilst some private nurses are not in regular employment. Each of these classes, in Mr. Ryan's opinion, will be unable to contribute £3 5s. per annum to a Pension Fund. It would be interesting, as Mr. Ryan says, to know what hospital officials and trained nurses in general think of the difficulties he alludes to. For our own part we believe the first objection will be found to apply mainly to midwives, a class by themselves; the third point has not been urged by any private nurse amongst the many who have sent in their names; and the second was anticipated and provided for in the scheme laid before the meeting on the 12th inst.

